# Ophthalmic nursing services in Botswana

**Published:** 2014

**Authors:** Chatawana Molao

**Affiliations:** Head of Advanced Ophthalmic Nursing: Molepolole Institute of Health Sciences, Molepolole, Botswana. chamolao@yahoo.com

**Figure:**
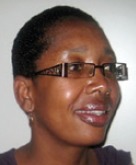
Chatawana Molao

Ophthalmic nurses make up 90% of all eye workers in Botswana's public sector, and they are the only eye professionals being trained inside the country.

In 2010, there were 88 ophthalmic nurses working in the public sector. The majority (74) were in primary and secondary care settings where they functioned independently under the supervision of general (non-ophthalmic) facility and department managers. Some received remote supervision and support from ophthalmologists based at tertiary hospitals, but this was minimal. Fourteen nurses worked in tertiary hospitals under the direct supervision of an ophthalmologist; they also scrubbed for minor and major ophthalmic surgical procedures.

Overall, most ophthalmic nurses (81%) worked in the urban or semi-urban areas inhabited by 61% of Botswana's approximately 2 million people. The 39% of people who live in the remote and isolated rural parts of the country were served by only 16 ophthalmic nurses (18%). Eye services remain a challenge in rural areas due to difficult terrain and limited financial and material resources. However, in many cases, ophthalmic nurses have built strong teams and share resources, such as transport, to extend services to all catchment areas within the district.

## Supervision and support

In all primary and district hospitals, the eye unit forms part of the outpatient department. The ophthalmic nurses are mostly managed and supervised by the head of the outpatient department or facility. In some units, this setup has caused conflict because non-ophthalmic managers are not aware of the importance of eye care services or have not fully understood the role and job description of the ophthalmic nurses.

Integration of ophthalmic services within mainstream health services is minimal, and efforts have begun to train general nurses and community-based health care assistants in primary eye care in order to strengthen collaborations and increase the availability of eye services.

## Referral pathways

Ophthalmic nurses form a central referral link for optometry services in both urban and rural settings, as they are the main public sector eye care providers. They are also the main contacts to whom primary health care workers refer patients. However, Botswana does not have a well-structured referral pathway for all health teams, particularly between the private and public sector. The absence of strong referral pathways from primary or district level to tertiary level means that eye patients who are referred for specialist eye care locally and externally cannot be easily traced in order to track their progress and monitor their condition closely. As a result of poor contact and communication, patients who are referred to centralised tertiary facilities do not take up these referrals. In some cases, patients who do take up referrals do not get to see the ophthalmologist they have been referred to. This is due to irregular opening times which are not communicated to patients. Another issue is the large proportion of ophthalmologists who resign or move on, due to the fact that they are usually employed on a contract basis.

This breakdown – or lack – of connection weakens the contact between the ophthalmic nurses, the ophthalmologists and the community.

## Challenges

A recent study^1^ identified several challenges.

- Working without the direct supervision of a specialist.
- The absence of a well documented scope of practice, professional registration and job description for ophthalmic nurses, which results in their being assigned to do general nursing.
- The fact that inexperienced ophthalmic nurses (most of whom are newly qualified) are sent to work in primary care settings where they are isolated and lack supervision.
- A lack of incentives to attract nurses and retain them in remote settings.
- Limited career pathways and professional development options.
- A lack of prevention of blindness policy guidelines, which means there is no governance or coordination of work done in the varied ophthalmic care settings.
- A lack of well defined or harmonised deployment and distribution of ophthalmic nurses in the public sector.

**Figure:**
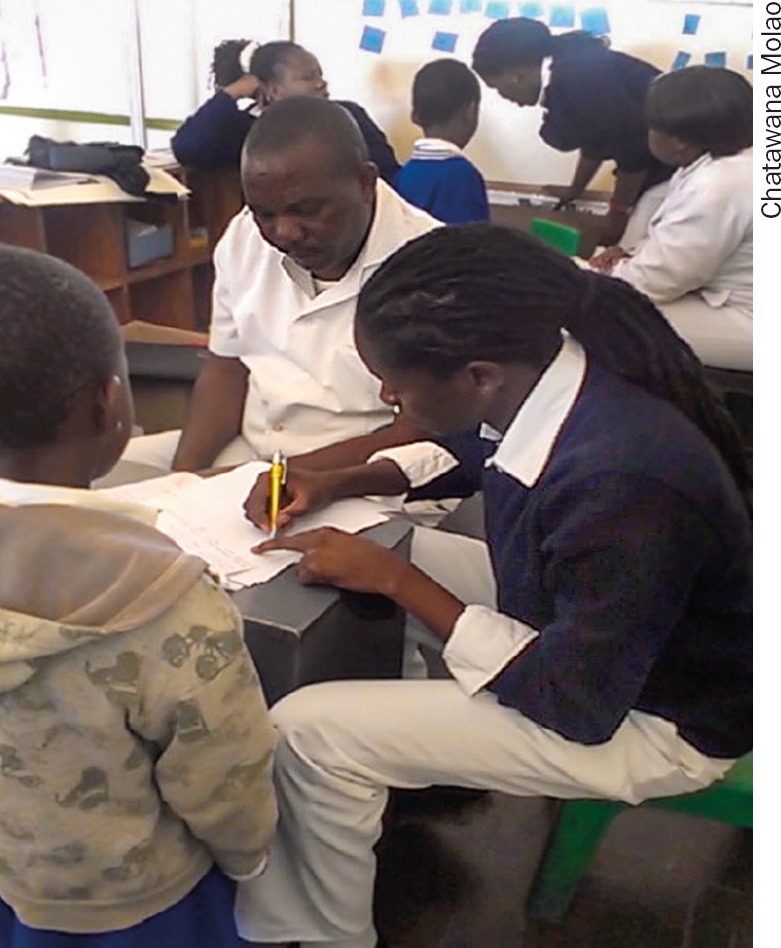
Ophthalmic nurses are involved in school health screening in Botswana

## Addressing the challenges

The Botswana Ministry of Health's prevention of blindness unit has established two tertiary centres of excellence and a VISION 2020 LINKS partnership with a UK institution (Addenbrookes Abroad, Cambridge) in an effort to improve eye care services in Botswana. Capacity building workshops have been organised through the partnership in order to enhance continuing professional development for ophthalmic nurses. Since 2013, about 17 ophthalmic nurses have been trained in basic refraction, 15 in diabetic retinopathy screening and 13 in children's vision screening. The training has strengthened networking among the ophthalmic nurses. They communicate quite often using communications technologies such as WhatsApp (an instant messaging application used on cell phones) and Facebook (a social networking platform) to share work experiences and support one another.

The ophthalmic nursing training programme at the Institute of Health Sciences (IHS) in Molepolole is currently reviewing its curriculum in an effort to upgrade the advanced diploma programme to an honours degree, and to increase enrolment from 12 to 15 candidates (every 2 years). It is also anticipated that the curriculum will include training in planning and managing eye care services.

Plans and advocacy to develop policy guidelines on the deployment and scope of practice of ophthalmic nurses are ongoing, and draft job descriptions have been developed to spell out their roles. This will mean that ophthalmic nurses will be better recognised and supported in their role within the broader health system.
